# OTC SENIOR STATION: CONSUMER-FACING TECHNOLOGY TO PROMOTE MEDICATION SAFETY

**DOI:** 10.1093/geroni/igad104.2208

**Published:** 2023-12-21

**Authors:** Jordan Hill, Aaron Ganci, Himalaya Patel, Noll Campbell, Michelle Chui, Ephrem Abebe, Richard Holden

**Affiliations:** Indiana University School of Public Health, Bloomington, Indiana, United States; Indiana University-Purdue University Indianapolis Herron School of Art + Design, Indianapolis, Indiana, United States; Richard L Roudebush VA Medical Center, Indianapolis, Indiana, United States; Purdue University College of Pharmacy, West Lafayette, Indiana, United States; University of Wisconsin-Madison, Madison, Wisconsin, United States; Purdue University College of Pharmacy, West Lafayette, Indiana, United States; Indiana University School of Public Health, Bloomington, Indiana, United States

## Abstract

Over-the-counter (OTC) medications with anticholinergic effects (e.g. doxylamine, diphenhydramine) are widely used by older adults, despite their association with an increased risk of Alzheimer’s disease and related dementias (ADRD). Authoritative bodies, such as the American Geriatrics Society and National Academy of Medicine, recommend discouraging the use of both prescription and OTC anticholinergics. However, most interventions previously developed and tested have targeted only prescription anticholinergics despite the use rate of OTC anticholinergics being higher in older adult populations. Consumer-facing technology may be ideal for addressing OTC anticholinergic use as it is cost effective, scalable, and can address the constraints of relying on busy pharmacists to intervene during older adult OTC medication purchases; this has become especially relevant as pharmacies face staffing shortages, increasing pharmacist workload and requiring pharmacies adjust their hours of operation. Our interdisciplinary team of pharmacists, engineers, and designers engaged both older adults and pharmacy staff in the co-design of “OTC Senior Station”: a user-centered, consumer-facing kiosk to be placed in community pharmacies to promote reduced anticholinergic use by older adults. Kiosk users are prompted to input brief information, which is used to present safer alternatives to OTC anticholinergics (e.g., for insomnia management). This poster will present OTC Senior Station--what we believe to be the first consumer-facing technology to target OTC anticholinergic use—the participatory design methods used to create it, and the results of studies to assess its usability with older adults.

